# Evaluation of Threshold Displacement Energies in InP Using Classical Molecular Dynamics

**DOI:** 10.3390/nano16171047

**Published:** 2026-08-22

**Authors:** Yurong Bai, Jiayu Liang, Shaowei He, Yonghong Li, Yang Li, Hang Zang, Fang Liu, Pei Li, Huan He, Chaohui He

**Affiliations:** 1 School of Nuclear Science and Technology, Xi’an Jiaotong University, Xi’an 710049, China; 2 Xi’an Rare Metal Materials Institute Co. Ltd., Xi’an 710016, China; 3 National Technology Innovation Center for Advanced Rare Metal Materials, Xi’an 710100, China; 4 Key Laboratory for Optical Measurement and Thin Films of Shaanxi Province, Xi’an Technological University, Xi’an 710041, China

**Keywords:** indium phosphide, threshold displacement energy, molecular dynamics

## Abstract

Benefiting from excellent high-frequency characteristics and superior radiation tolerance, InP is an indispensable material for next-generation high-speed communications, widely applied in optical communication, 6G radio frequency chips, AI optical interconnection, and aerospace radiation-hardened electronics. Although ion implantation greatly promotes the performance optimization of InP-based devices, it inevitably induces lattice displacement defects that degrade device reliability. Hence, quantitative evaluation of the threshold displacement energy (TDE) and dominant defect configurations in InP is essential. Our calculations reveal that the average threshold displacement energy is 18.20 eV for In atoms and 18.94 eV for P atoms. The *E*_d_ distributions for both In and P atoms predominantly lie below 30 eV and rarely exceed 40 eV. From 150 K to 900 K, In and P have a large mass difference and exhibit distinct temperature-dependent trends. The threshold displacement energy of In decreases with increasing temperature, whereas that of P rises as temperature increases. Based on the structural analysis of Frenkel pairs formed by displaced atoms, the dominant interstitial configurations are identified. These results provide detailed insights for damage evaluation and defect structure characterization in InP, benefiting ion implantation process optimization and radiation-hardening design of InP electronic devices.

## 1. Introduction

As a representative III–V semiconductor, InP features excellent radiation hardness, high electron mobility, and high electron saturation drift velocity [1], outperforming Si, GaAs, and GaN [2]. Accordingly, it serves as an indispensable material for next-generation high-performance devices deployed in high-speed optical communication, AI computing, 6G millimeter-wave radiofrequency devices and aerospace radiation-hardened electronics. However, the operational stability and long-term reliability of InP-based devices are significantly hindered by radiation-induced defects [3,4,5,6,7,8,9,10,11,12]. When exposed to high-energy particles (e.g., protons, electrons, and heavy ions) in during ion implantation or in the harsh space radiation environment, lattice atoms in InP can be displaced from their equilibrium positions, leading to the formation of vacancies and interstitials. These defects severely degrade the carrier transport properties and structural integrity of the material [13,14,15], thereby degrading device performance.

Although the radiation effects of InP have attracted extensive attention, the key microscopic parameters related to primary radiation damage remain insufficiently studied. In particular, the threshold displacement energy (*E*_d_) of InP, which is the minimum energy required to displace lattice atoms, has not been calculated. *E*_d_ is a critical parameter for evaluating radiation damage induced by different particles using the NRT [16] and arc-dpa [17] models. Since experimental measurement of atomic displacement occurring on the picosecond (ps) time scale is challenging, theoretical calculations can provide detailed atomic-scale evolution processes of defects and are thus successfully employed to accurately calculate the *E*_d_ of semiconductor materials.

Molecular dynamics (MD) is widely adopted to calculate *E*_d_ [18,19,20,21,22,23,24], and the accuracy of MD simulations strongly depends on the selection of a suitable interatomic potential [25,26]. Branicio et al. [27] developed the Vashishta potential for InP to predict its thermal and mechanical properties. However, this potential shows limited performance in describing covalent bond breaking during particle collisions. The analytical bond-order potential (ABOP) is physically more consistent with the bonding characteristics of covalent semiconductors and can compensate for the shortcomings of the Vashishta potential [28,29]. The ABOP for InP constructed by Chrobak et al. [30] can simulate the B3↔B1 phase transition and the formation energy of point defects, but it tends to underestimate the short-range repulsive interactions between atoms. Recently, we introduced the Ziegler–Biersack–Littmark (ZBL) [21] potential to describe the strong short-range repulsion during high-energy atomic collisions and constructed an ABOP+ZBL [31] potential for InP. Systematic calculations including defect formation energy, quasi-static property tests, and primary radiation damage simulations demonstrate that the ABOP+ZBL potential can reliably describe the atomic interactions in InP.

In this work, we use the ABOP+ZBL potential to calculate *E*_d_ of InP over 4000 atomic trajectories along different directions and obtain its spatial distribution. We also investigate the influence mechanism of temperature (150~900 K) on *E*_d_ and systematically analyze the defect configurations induced by recoil atoms in InP. These fundamental data elucidate the intrinsic radiation damage mechanisms of InP, providing significant guidance for ion implantation optimization and radiation-hardened design of InP-based devices.

## 2. Methodology

**Figure 1:**
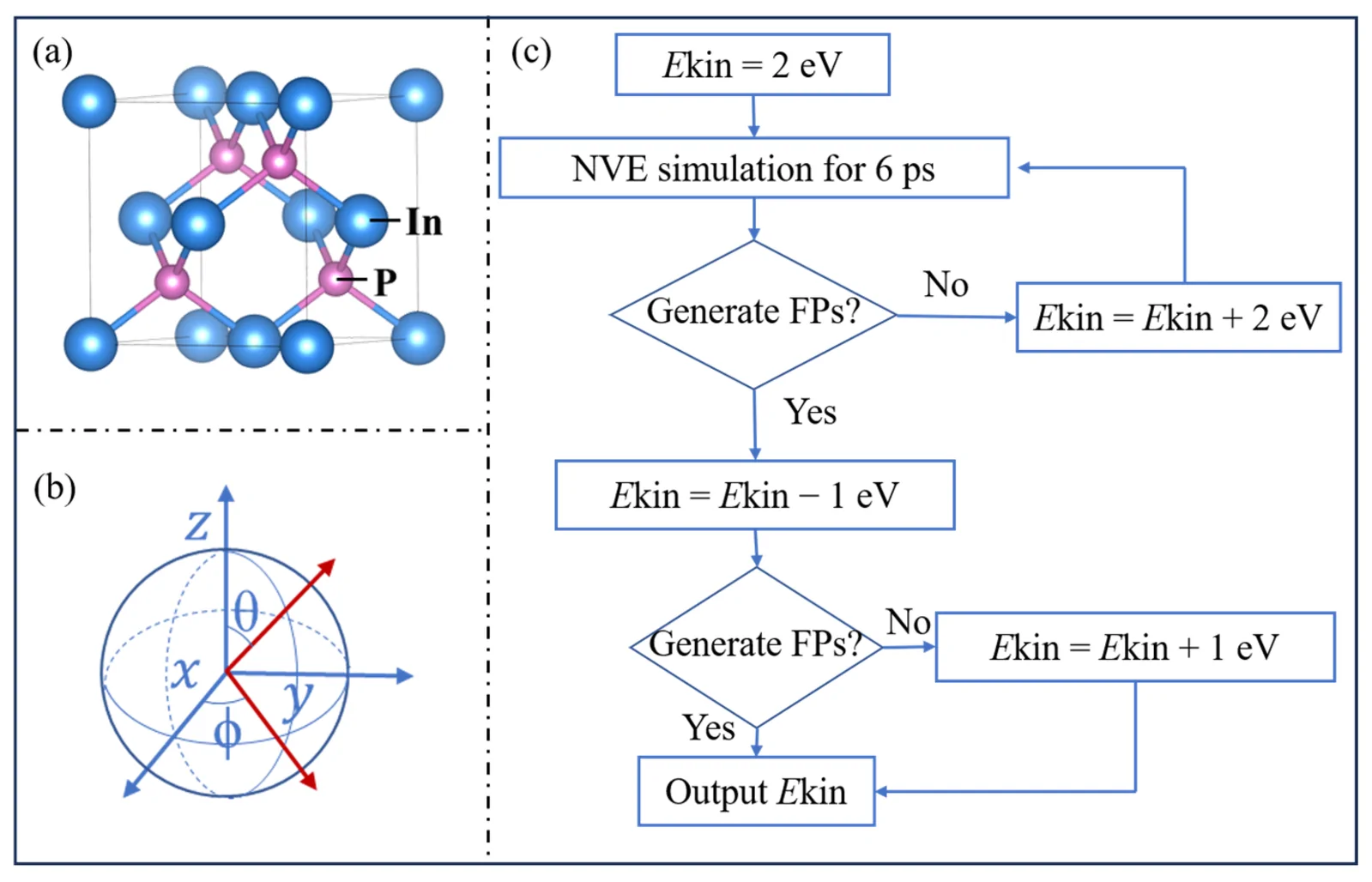
Simulation setup: (**a**) Crystalline structure of InP. (**b**) the Spherical Coordinate System, the red arrows represent the emission directions of recoil atoms. (**c**) Flow chart of threshold displacement energy calculation.

All threshold displacement energy simulations for In and P recoil atoms in InP are calculated by using the Large-scale Atomic/Molecular Massively Parallel Simulator (LAMMPS, v2023) [32] code. A simulation supercell consisting of 10 × 10 × 10 unit cells (8000 atoms, with a lattice constant of 5.869 Å) is constructed, with a zinc-blende InP structure, as depicted in Figure 1a. To accurately capture the high-energy recoil events and subsequent defect evolution during TDE calculations, the ABOP is smoothly coupled with the ZBL universal repulsive potential via a Fermi-type switching function, where the switching parameters for In-In, In-P, and P-P interactions are set to (14.5 Å^−1^, 0.99 Å), (14.5 Å^−1^, 0.99 Å), and (7.8 Å^−1^, 0.67 Å), respectively, as detailed in Ref. [31]. At finite temperatures, thermal fluctuations and initial atomic velocity distributions [33] (determined by velocity seeds) can introduce statistical uncertainty into the calculated *E*_d_ values. To mitigate this stochasticity, the system is first equilibrated in a canonical (NVT) ensemble for 5 ps to achieve the thermal equilibrium. Three snapshots are extracted at 0.5 ps intervals as initial configurations. Two velocity seeds are assigned for each snapshot, yielding six initial configurations in total. The calculated average threshold displacement energies from six initial configurations at 300 K are summarized in Table S1. Slight perturbations in the computed threshold-displacement energy are introduced by different atomic configurations and initial velocity seeds.

During the atomic collision simulations, the microcanonical (NVE) ensemble is maintained. The simulation box is geometrically partitioned into an inner region and an outer boundary layer with a thickness of 5.869 Å. To prevent the primary knock-on atom (PKA) from crossing the periodic boundaries during the cascade, a single atom (In or P) is randomly selected as the PKA strictly from the inner region. As illustrated in Figure 1b, the emission direction of the primary knock-on atom (PKA) is uniformly sampled over the spherical surface. The polar angle *θ* is obtained by inverse-transform sampling $\theta =\arccos(1-2r_{1})$, and the azimuthal angle $\phi =2\pi r_{2}$, where $r_{1}$ and $r_{2}$ are two independent uniform random numbers from 0 to 1. This scheme avoids the pole-clustering bias originating from direct uniform sampling of *θ* and ϕ, and ensures isotropic uniform sampling across the unit-sphere surface [34].

The threshold search procedure is initiated by assigning the PKA an initial kinetic energy (*E*_kin_) of 2 eV as shown in Figure 1c. This energy is systematically incremented by 2 eV per iteration until the formation of a stable Frenkel pair (FP) is observed. To pinpoint the precise threshold, a refined search is then conducted by decreasing the knock-on energy by 1 eV to determine the ultimate *E*_d_. Each TDE value has an uncertainty of ±1 eV, as trial recoil energies are increased in 2 eV steps. To ensure the complete stabilization of the generated defects, each collision cascade is allowed to evolve for 6 ps using an adaptive time step of ~1 fs. Finally, the point defects generated during the cascades are identified utilizing the modified Wigner–Seitz cell method [35]. The specific interstitial structures are further classified based on the atomic coordination number calculated within 3.5 Å.

To calculate *E*_d_, we carry out the MD simulations of different types of PKA in approximately 6000 directions, each type of PKA is simulated over 3000 random directions. The average TDE (*E*_ave_) over all directions for each atom is calculated according to the formula [36]:

(1) $$E_{ave}=\frac{\iint E(\theta ,\phi )\sin(\theta )d\theta d\phi }{\iint \sin(\theta )d\theta d\phi }$$

where $\theta \in (0^{{}^{\circ}},180^{{}^{\circ}})\;$ and $\phi \in (0^{{}^{\circ}},360^{{}^{\circ}})$.

Convergence tests of *E*_ave_ under different system sizes (from 4096 atoms to 64000 atoms) and cascade simulation durations (from 6 ps to 10 ps) are presented in Figures S1 and S2. With over 1000 independent TDE simulation runs, the calculated *E*_ave_ values exhibit good consistency across different system sizes and cascade durations, and the standard error of mean (SEM) is kept below 0.16 eV.

## 3. Results and Discussion

### 3.1. Threshold Displacement Energy

**Figure 2:**
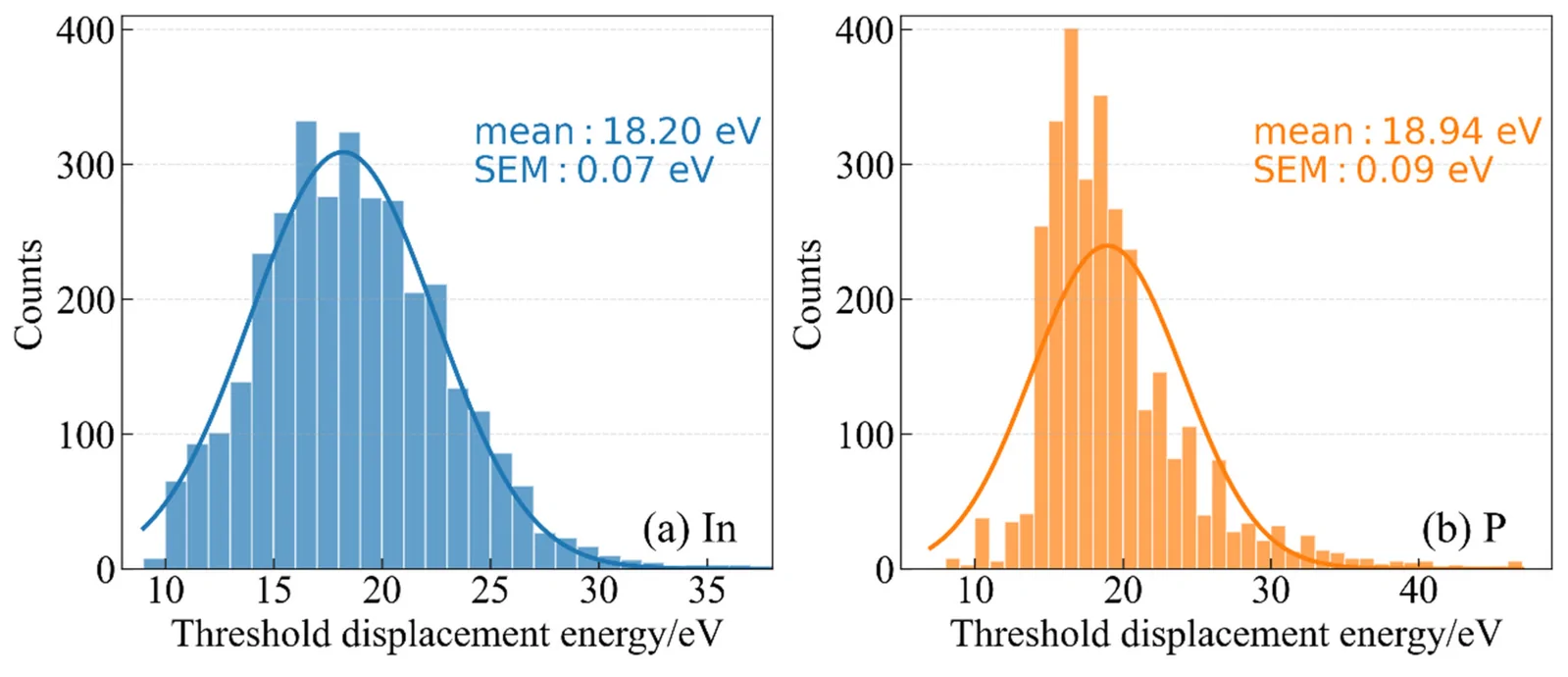
Threshold displacement energies for In and P.

The distribution of threshold displacement energy for In atoms and P atoms is shown in Figure 2. The averaged threshold displacement energies are 18.20 eV for In and 18.94 eV for P, with the SEM for both species less than 0.1 eV, which confirms that the number of recoil simulations is statistically sufficient to produce reliable results.

Threshold displacement energies exhibit anisotropic behavior in crystalline materials, and the calculated threshold displacement energies along typical crystallographic directions of InP are listed in Table 1. The calculated global minimum *E*_d_ for P is 8 eV, which exhibits excellent agreement with the macroscopic effective threshold of 8.7 eV [37], demonstrating the foundational reliability of the simulations. Conversely, for In atoms, the global minimum *E*_d_ (9 eV) is higher than the macroscopic experimental value of 6.7 eV [37]. This numerical deviation arises because the classical MD framework strictly evaluates the theoretical upper limit of the ground-state elastic collision barrier. It inherently lacks the capability to capture the barrier-lowering effects induced by non-adiabatic ionization mechanisms prevalent in actual electron irradiation experiments [38]. While first-principles methods can capture these non-adiabatic processes, their immense computational cost restricts simulations to small supercells and tens-of-femtosecond timescales, precluding the exhaustive angular sampling required to systematically map the anisotropic distribution of *E*_d_. The calculated minimum *E*_d_ for both In (18 eV and 9 eV) and P (13 eV and 12 eV) along [111] and $\left[\bar{1}\bar{1}\bar{1}\right]$ directions exceed experimental measurements, but the predicted trend agrees well with experimental observations. Therefore, MD simulations remain indispensable, as they provide the required spatiotemporal scale for statistically evaluating the complex directional dependence of *E*_d_ and comprehensively uncover the atomic mechanisms behind defect generation in InP.

**Figure 3:**
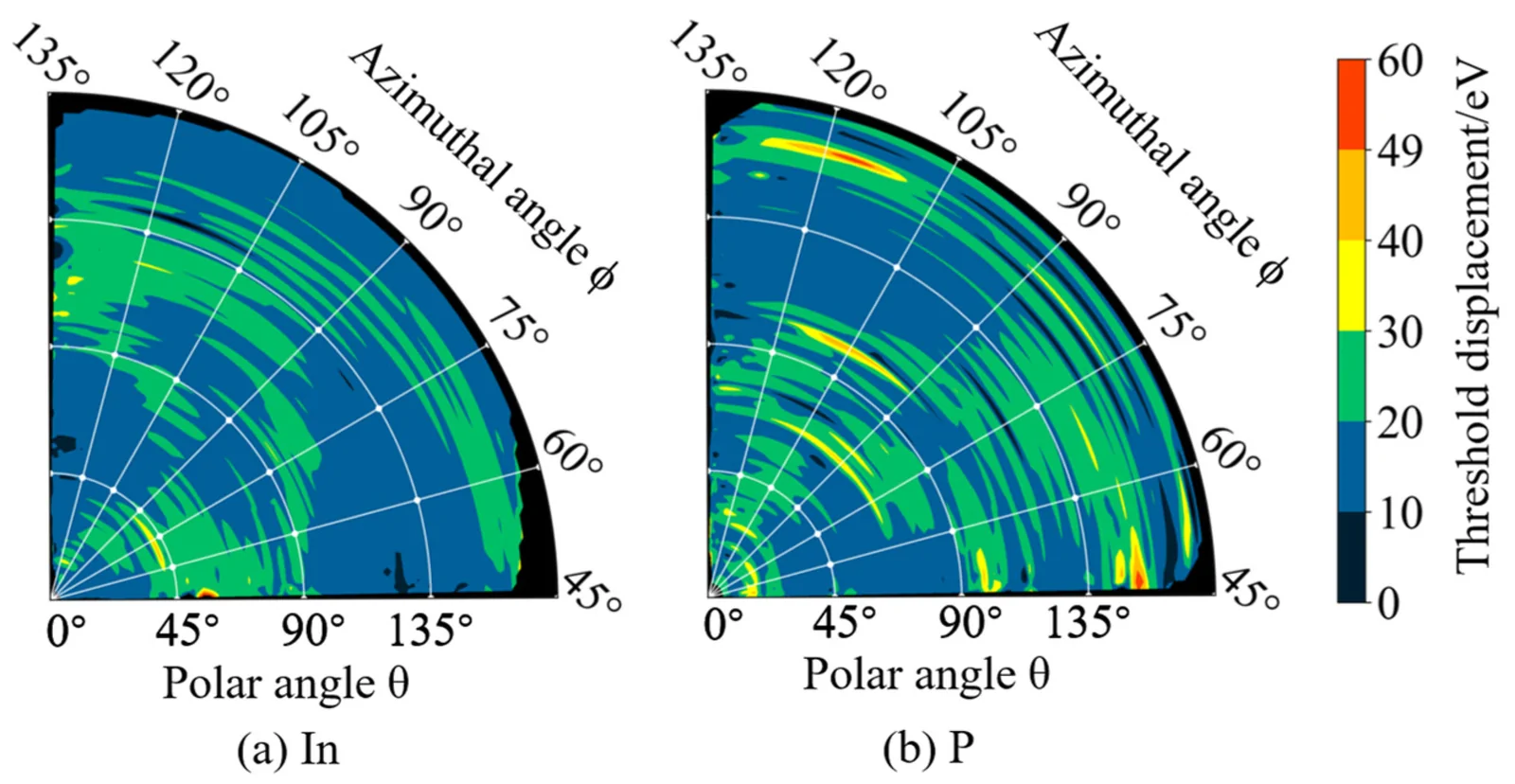
Angular maps of the threshold displacement energies in InP.

Considering the significant influence of recoil direction on *E*_d_ [24,40], the spatial distribution of *E*_d_ for In and P atoms in InP is analyzed in Figure 3. Owing to the cubic symmetry of zinc-blende InP, equivalent azimuthal angles are folded into a representative 45°~135° sector using a mirror and 180-degree rotation operations at a constant polar angle. The symmetry-mapped directions are crystallographically equivalent to the original ones, allowing the reduced-sector data to fully represent the angular dependence of *E*_d_. The results show that the distribution mechanisms of threshold displacement energy for In and P atoms are similar. As depicted in Figure 3a, the *E*_d_ landscape for In atoms is predominantly characterized by lower energy values (green regions, typically below 30 eV) and exhibits a relatively smooth, homogeneous angular dependence. In stark contrast, the *E*_d_ map for P atoms (Figure 3b) displays pronounced crystallographic anisotropy, evidenced by distinct, narrow high-energy streaks and localized hot spots (yellow and red regions exceeding 40 eV) that are prominently distributed at specific orientations, particularly at larger polar angles. This obvious macroscopic difference directly reflects the underlying collision physics. During the ballistic phase, light P atoms suffer strong directional scattering and geometric hindrance by the heavier In sub-lattice. Compared with heavier In atoms, P atoms exhibit highly direction-dependent energy barriers for stable Frenkel defect generation.

**Figure 4:**
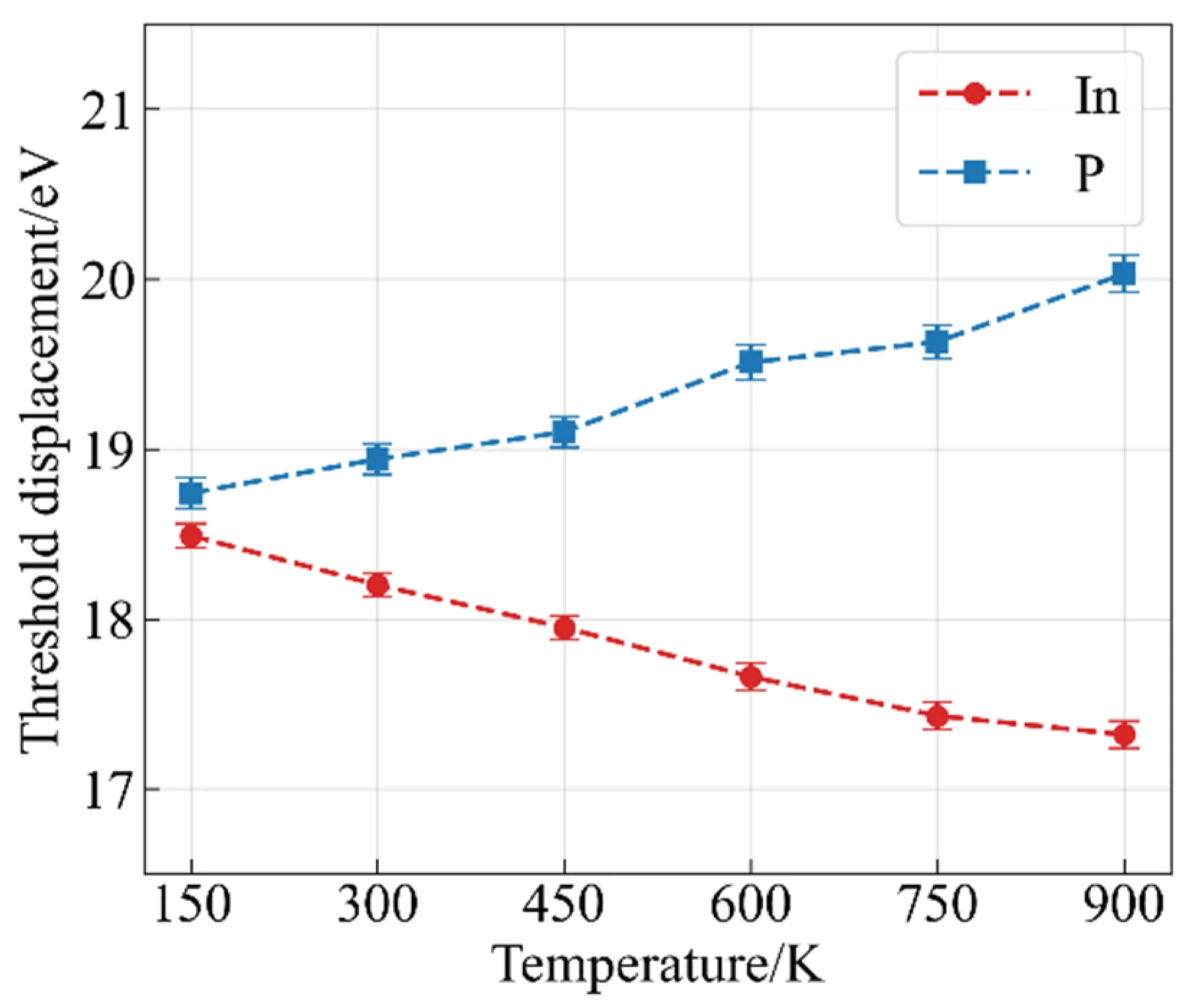
Temperature dependence of the average threshold displacement energy for In and P atoms in InP. Error bars show the standard errors of the mean (SEM).

To explore the behavior of InP in the extreme-temperature environment of space, the variation trend of the average threshold displacement energy of InP from 150 K to 900 K is presented in Figure 4. From 150 K to 900 K, the *E*_ave_ of In atoms decreases from 18.49 eV to 17.32 eV, whereas that of P atoms increases from 18.74 eV to 20.03 eV. This divergent temperature dependence fundamentally arises from the interplay between defect migration–recombination dynamics and thermal expansion effects within the lattice. This opposite temperature behavior originates from the competition between defect migration-recombination and lattice thermal expansion. For In atoms, the high migration barrier (1.21 eV [41]) suppresses spontaneous defect recombination. Thus, lattice thermal expansion dominates, reducing the geometric escape barrier and lowering *E*_ave_. In comparison, ejected light P atoms undergo strong scattering by thermally vibrating heavy In atoms, which increases kinetic-energy loss. Higher initial energies are therefore required for P atoms to leave the lattice, so *E*_ave_ rises at higher temperatures.

### 3.2. Defect Configurations

**Figure 5:**
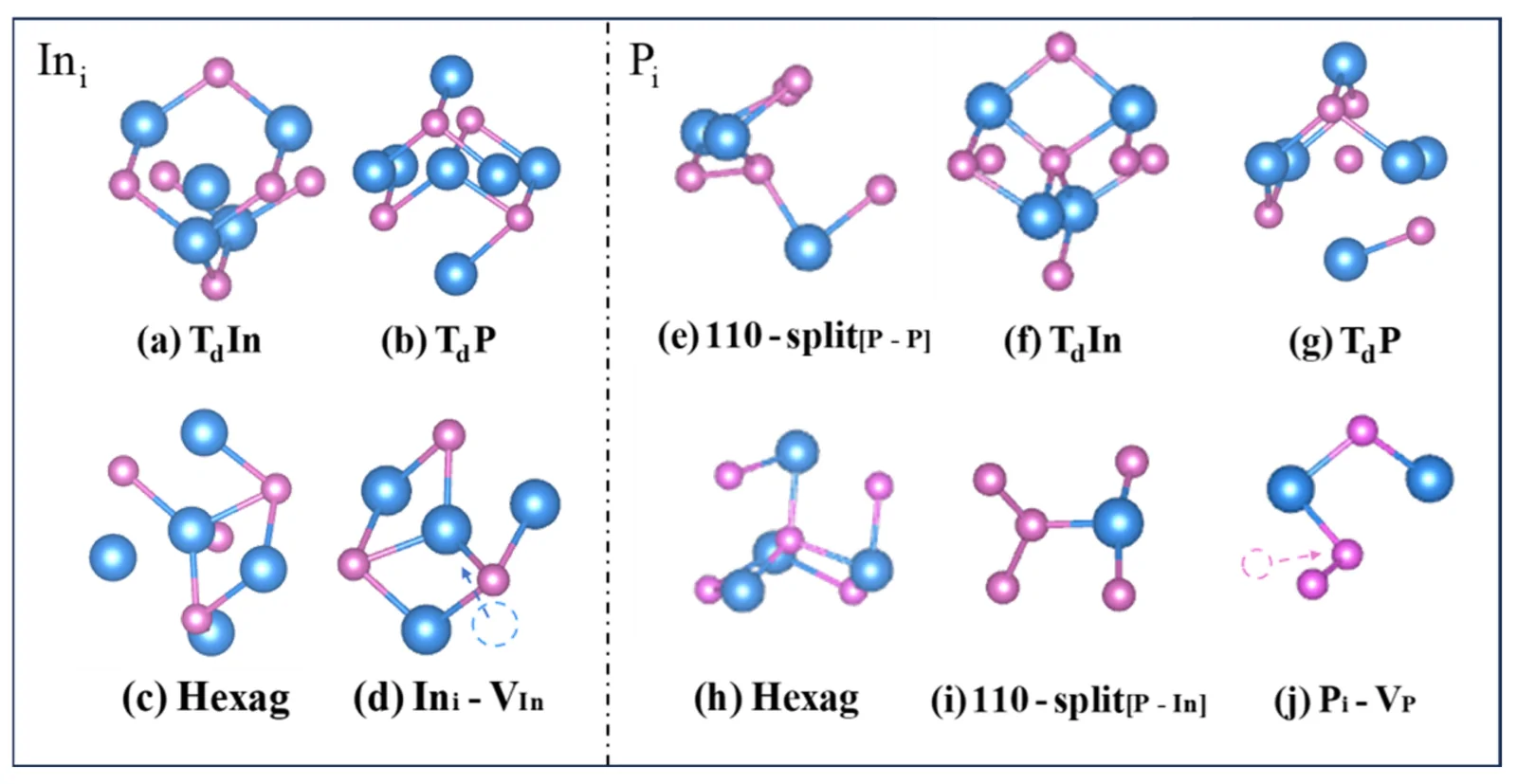
The atomic configurations of (**a**–**d**) In_i_ and (**e**–**j**) P_i_.

Since vacancy-interstitial pairs (Frenkel pairs, FPs) are the primary radiation-induced defects, we classify the configurations of FPs from 6365 threshold displacement energy simulations to explore their microscopic characteristics. In total, 6405 interstitial defects are generated across all simulations, including 3742 Inᵢ and 2663 Pᵢ. The typical atomic configurations [42,43,44] of In_i_ and P_i_ in InP are shown in Figure 5. Among these, tetrahedral defect T_d_In refers to an interstitial located at the center of a tetrahedron formed by four In atoms. Tetrahedral defect T_d_P refers to an interstitial located at the center of a tetrahedron formed by four P atoms. Hexag indicates an interstitial positioned at the center of six atoms (a hexahedral site). X_i_-V_X_ denotes an interstitial X located relatively close to a lattice site, forming a Frenkel pair with a vacancy. For P_i_, 110-split_[P-P]_ represents a P-P dumbbell interstitial along <110>, while 110-split_[P-Pn]_ represents a P-In dumbbell interstitial along <110>.

We statistically analyze defect proportions, as shown in Table 2, to explore the structural stability of interstitials in InP. For Inᵢ, the defects predominantly occupy tetrahedral interstitial sites, with the Inᵢ-T_d_P (57.78%) and Inᵢ-T_d_In (39.07%) configurations comprising being overwhelmingly dominant. This indicates that In atoms are more susceptible to displacement and tend to form complex interstitial defects. In contrast, P_i_ are less susceptible to displacement and tend to bond with next-nearest-neighbor P atoms, dominated by the Pᵢ-V_P_ configuration (46.10%) and the 110-split_[P-P]_ configuration (36.37%).

**Figure 6:**
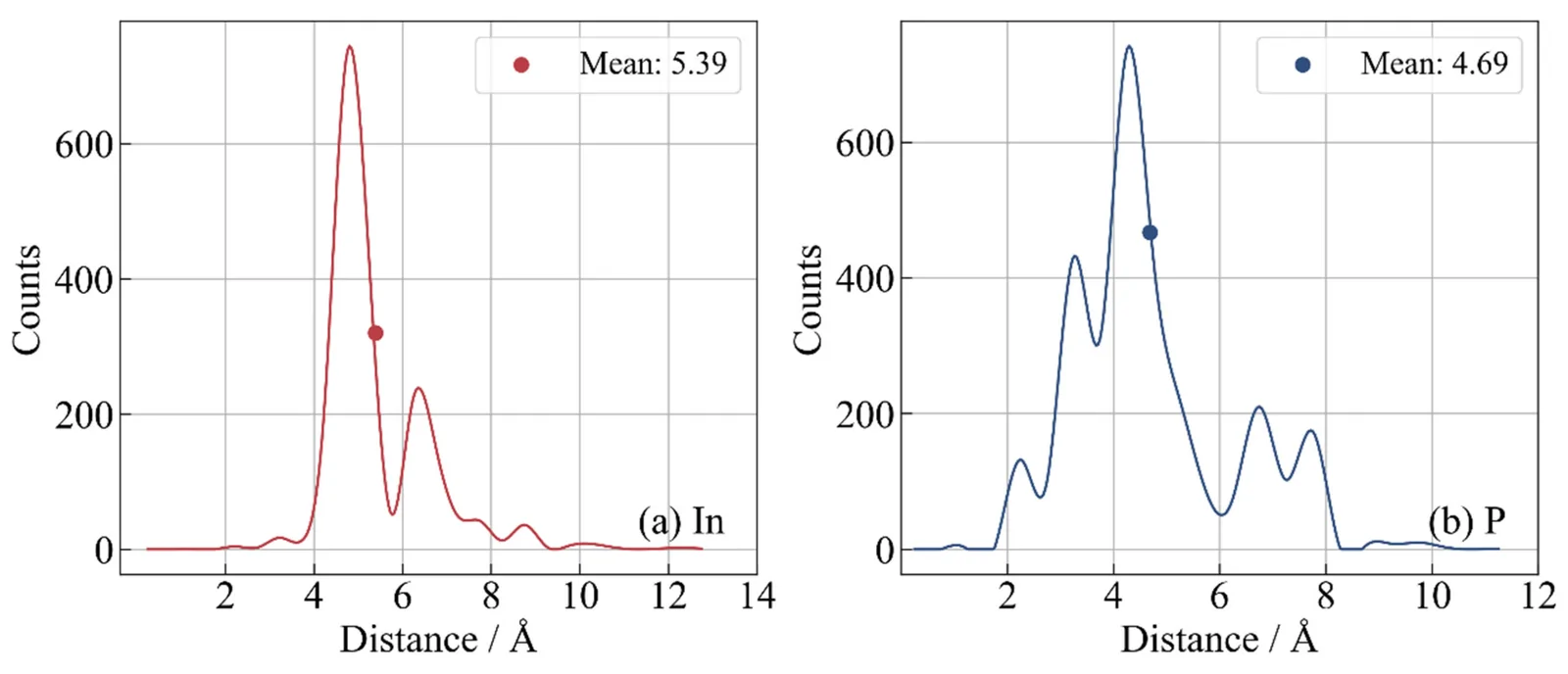
Maximum distance of FPs before and after relaxation: (**a**) In_i_-V_In_; (**b**) P_i_-V_P_.

To investigate interstitial diffusion behavior, the maximum distances between vacancies and interstitials are presented in Figure 6. The figure shows that Inᵢ and Pᵢ can diffuse from their initial positions up to 8 Å, which may cause local stress and influence the electronic properties of InP [44,45]. 

## 4. Conclusions

In this work, MD simulations are performed to calculate the threshold displacement energies of InP. The results indicate that the average *E*_d_ values for In and P are 18.20 eV and 18.94 eV, respectively. The minimum *E*_d_ for P exhibits excellent consistency with experimental data. Although the minimum *E*_d_ for In overestimates the experimental value because classical MD frameworks inherently lack the capability to capture non-adiabatic ionization processes, the predicted anisotropic angular distribution aligns perfectly with experimental trends. This confirms the foundational reliability of the simulations. The *E*_d_ distributions for both In and P atoms predominantly fall below 30 eV and rarely exceed 40 eV. Temperature-dependent calculations from 150 K to 900 K reveal that the *E*_ave_ of In decreases at elevated temperatures, whereas the *E*_ave_ of P increases. This divergent behavior is attributed to the fact that at high temperatures, In atoms are more easily displaced from the surrounding lighter P atoms, while P atoms encounter greater difficulty in escaping from the heavier In atoms. Statistical analysis on the proportion of defect configurations induced by displaced atoms reveals that In_i_-T_d_In, In_i_-T_d_P and P_i_-110-split_[P-P]_ are dominant configurations. Furthermore, the maximum distances between FPs for both In and Pi defects are observed to be approximately 8 Å. These calculations provide valuable insights into the radiation damage and the complex defect structures in InP.

## Figures and Tables

**Table 1:** Overview of *E*_d_ in InP compared with experiment data. Min denotes the minimum value of *E*_d_.

Directions	*E*_d_(In)/eV			*E*_d_(P)/eV		
	Average	Min	Experiment	Average	Min	Experiment
all	18.20	9	6.7 [37]	18.94	8	8.7 [37]
[111]	22.56	18	4 [39]	15.57	13	8 [39]
$\left[\bar{1}\bar{1}\bar{1}\right]$	9.93	9	3 [39]	15.41	12	8 [39]
[100]	19	13	-	17.88	8	-
[110]	17.34	12	-	18.63	12	-

**Table 2:** Summary of interstitial coordination numbers in InP. Coordination numbers (a, b) represent the counts of In and P atoms within a cutoff radius of 3.5 Å for interstitial atoms.

Defect		Ideal	Non-Ideal	Proportion/%
In_i_	T_d_In	(4,6)	(3,7), (4,5), (3,6), (3,4)	39.07
	T_d_P	(6,4)	(7,3), (5,4), (5,3), (4,3)	57.78
	Hexag	(4,4)	(4,3)	1.39
	In_i_-V_In_	≤6	-	1.76
	110-split_[P-P]_	(4,3)	(3,4), (4,2), (2,4), (3,3), (2,3), (3,2)	36.37
P_i_	T_d_In	(4,6)	(3,7), (4,5), (5,4), (3,6), (2,7), (3,5), (6,2), (2,5), (5,1)	14.18
	T_d_P	(6,4)	(7,3), (4,3), (4,2)	0.08
	Hexag	(4,4)	-	2.03
	110-split_[P-In]_	(1,4)	-	1.24
	P_i_-V_P_	≤4	-	46.10

## Data Availability

All data used in this study are available upon request from the corresponding author.

## Supplementary Materials

The following supporting information can be downloaded at: https://www.mdpi.com/article/10.3390/nano16171047/s1. Table S1. Average displacement threshold energies (Eave) of In and P atoms in InP at 300 K under different thermal configurations and velocity seeds. Figure S1. Convergence of the average threshold displacement energy for InP with different supercell sizes, shown as the cumulative moving averages as functions of the number of simulated directions. Figure S2. Convergence of the average threshold displacement energy in InP for different cascade collision times, shown as the cumulative moving averages as functions of number of simulated directions.
