# Supplementary material for: Evaluation of Threshold Displacement Energies in InP Using Classical Molecular Dynamics
Source: Nanomaterials (Basel). 2026 Aug 22;16(17):1047. doi: 10.3390/nano16171047 (PMC13567601; doi:10.3390/nano16171047)
Supplement: Supplementary file 1 [file nanomaterials-16-01047-s001.zip › nanomaterials-4484679-supplementary.pdf]

# Supplementary Material

## Evaluation of threshold displacement energies in InP using classical molecular dynamics

Yurong Bai [1,2,3], Jiayu Liang [1], Shaowei He [1], Yonghong Li [1], Yang Li [1], Hang Zang [1], Fang Liu [1,4], Pei Li[1,*], Huan He[1,*], Chaohui He[1,*]

1 School of Nuclear Science and Technology, Xi’an Jiaotong University, Xi’an 710049, China;

2 Xi’an Rare Metal Materials Institute Co. Ltd, Xi’an, 710016, China

3 National Technology Innovation Center for Advanced Rare Metal Materials, Xi'an 710100, China.

4 Key Laboratory for Optical Measurement and Thin Films of Shaanxi Province, Xi'an Technological University, Xi’an, 710041, China

[*]Authors to whom correspondence should be addressed: lipei0916@xjtu.edu.cn; huanhe@xjtu.edu.cn; hechaohui@xjtu.edu.cn;

Table S1. Average displacement threshold energies ($E_{ave}$) of In and P atoms in InP at 300 K under different thermal configurations and velocity seeds.

| Thermal configurations | Velocity seeds | $E_{ave}$/eV | |
|---|---|---|---|
| | | In | P |
| 1 | 1 | 18.20±0.18 | 18.94±0.21 |
| 1 | 2 | 18.17±0.19 | 19.15±0.23 |
| 2 | 1 | 18.31±0.19 | 18.78±0.23 |
| 2 | 2 | 18.20±0.19 | 18.88±0.22 |
| 3 | 1 | 18.09±0.18 | 18.82±0.22 |
| 3 | 2 | 18.25±0.19 | 19.07±0.24 |

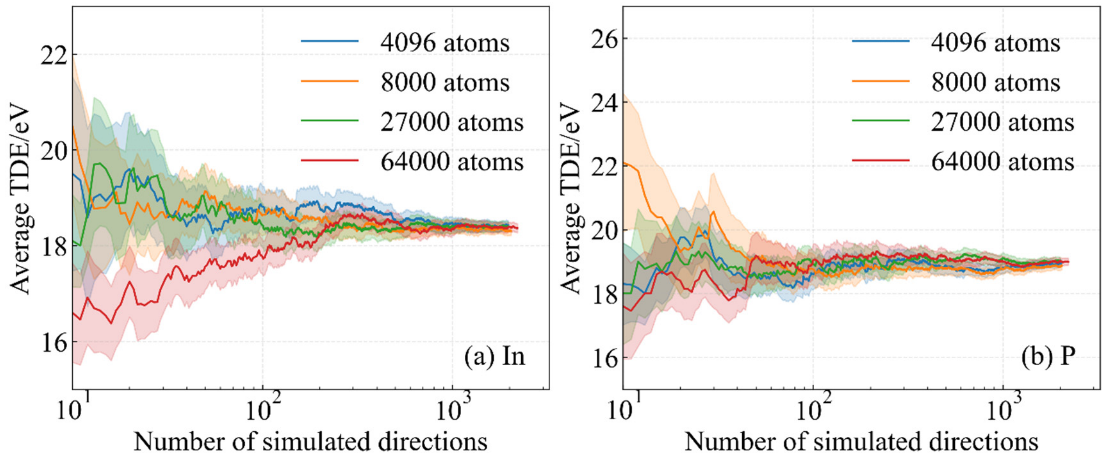


Figure S1 Convergence of the average threshold displacement energy for InP with different supercell sizes, shown as the cumulative moving averages as functions of the number of simulated directions. The shaded areas denote the standard errors of the mean (SEM).

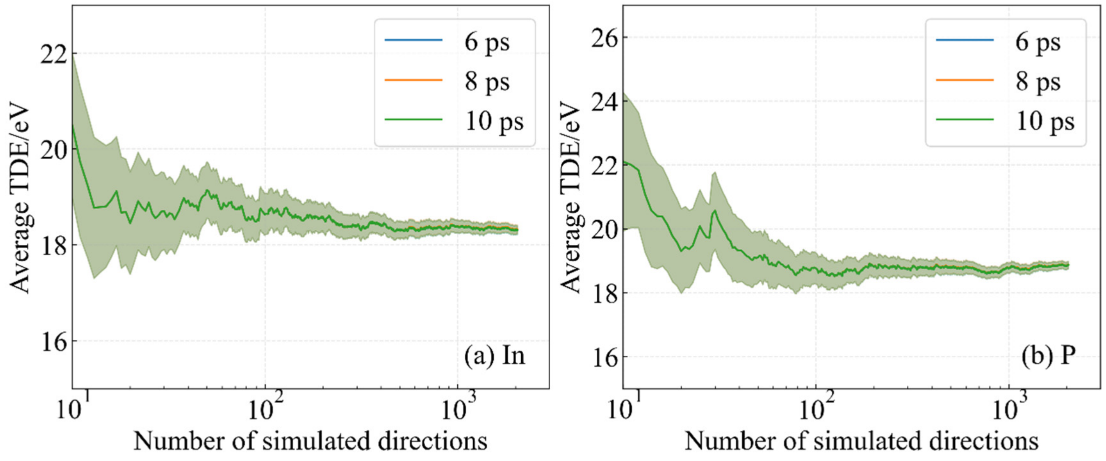


Figure S2 Convergence of the average threshold displacement energy in InP for different cascade-collision times, shown as the cumulative moving averages as functions of number of simulated directions. The shaded areas show the standard errors of the means (SEM).
